# Monoclonal Interstitial Nephritis with IgA-Kappa Plasma Cells Deposition in Sjögren Syndrome: A Case Report

**DOI:** 10.1159/cnd/abxag004

**Published:** 2026-09-08

**Authors:** Mario Alamilla-Sanchez, Nicte Alaide Ramos García, Stiven Cardenas Hurtado, Manuel Camacho Luna, Citlali De la Torre García, Regina Canade Hernández Hernández, Carolina Martínez Mijares

**Affiliations:** a Department of Nephrology, Centro Médico Nacional “20 de Noviembre”, Mexico City, Mexico; b Department of Pathology, Centro Médico Nacional “20 de Noviembre”, Mexico City, Mexico

**Keywords:** Tubulointerstitial nephritis, Monoclonal gammopathy of renal significance, Sjögren syndrome, IgA paraproteinemia, Plasma cell

## Abstract

**Introduction:**

In Sjögren syndrome, tubulointerstitial involvement is a frequent finding, characterized by lymphoplasmacytic infiltration, tubular atrophy, and interstitial fibrosis. Nevertheless, descriptions of monoclonal interstitial infiltrates remain scarce. These observations suggest that beyond the classical spectrum of glomerular or tubular injury, a subset of patients with Sjögren syndrome may present atypical renal manifestations potentially associated with underlying monoclonal gammopathy of renal significance. This highlights the importance of accurately characterizing infiltrates and carefully considering monoclonal gammopathy of renal significance (MGRS) as a potential contributor to disease heterogeneity and long-term kidney outcomes.

**Case Presentation:**

An 83-year-old woman with Sjögren syndrome presented with a rapidly progressive decline in kidney function, without proteinuria. Immunologic and viral workup was unremarkable. A kidney biopsy revealed marked interstitial plasma cell infiltration, as demonstrated by CD138+ staining and IgA-kappa restriction by immunohistochemistry, with no glomerular involvement. Bone marrow evaluation showed <10% plasma cells, excluding multiple myeloma. Serum electrophoresis confirmed an IgA-kappa monoclonal spike and abnormal free light chain ratio. Chemotherapy with bortezomib, dexamethasone, and lenalidomide resulted in an improvement of kidney function and normalization of IgA levels.

**Conclusion:**

The interstitium is frequently overlooked in MGRS classification; however, interstitial monoclonal plasma cell infiltration has recently been described in a subgroup of patients with Sjögren syndrome. It may represent a novel entity within MGRS, potentially enhancing prognosis through plasma cell-directed therapies.

## Introduction

Sjögren syndrome is a chronic autoimmune disorder mainly affecting exocrine glands, but up to one-third of patients develop kidney involvement, mainly tubulointerstitial nephritis, which leads to diverse clinical signs and variable renal outcomes. New evidence suggests a possible overlap with monoclonal gammopathy of renal significance (MGRS), raising questions about shared disease mechanisms and diagnostic challenges.

The International Kidney and Monoclonal Gammopathy Research Group (IKMG) defines monoclonal gammopathy of renal significance (MGRS) as the presence of monoclonal immunoglobulin less than 3 g/dL and fewer than 10% clonal plasma cells in the bone marrow, associated with one or more kidney lesions related to the monoclonal immunoglobulin produced. Additionally, the cloning of plasma cells or B cells does not cause tumor complications or meet any hematological criteria for specific therapy [1].

The strongest independent predictors for detecting MGRS in biopsy specimens are a high proportion of free light chains (FLCs) in the serum, proteinuria of at least 1.5 g/day, and hematuria. Regarding hematological predictors, increased FLCs are associated with a higher risk of kidney injury [2].

Although reduced kidney function or proteinuria is usually detected in most patients, there is still no well-defined consensus on when to start specific chemotherapy treatment for many patients in whom the circulating pathological clone is not detected. The treatment for many cases of MGRS remains uncertain; therefore, a collaborative evaluation with a hematologist is essential.

We report a case of a patient with Sjögren syndrome who experienced rapid decline in kidney function without proteinuria and a serum monoclonal IgA spike. The kidney biopsy revealed interstitial deposits of IgA-kappa plasma cells, with no significant glomerular alterations or bone marrow infiltration. In collaboration with hematologists, a decision was made to initiate chemotherapy, which resulted in improved kidney function and no signs of disease progression.

## Clinical Case

An 83-year-old woman was referred to the nephrology outpatient clinic due to rising serum creatinine (sCr) levels and edema. She experienced muscle weakness, severe fatigue, unintentional weight loss, and intermittent fever over 2 months. Her relevant medical history included a diagnosis of Sjögren syndrome 1 year prior, managed with hydroxychloroquine and artificial tears, with regular follow-up at the rheumatology outpatient clinic. During the physical exam, mild pallor, absence of cardiac murmurs, and moderate limb edema were noted. Blood pressure and heart rate were normal. Laboratory tests revealed anemia and a serum creatinine (sCr) level of 3.2 mg/dL (baseline: 0.6 mg/dL 3 months earlier), blood urea nitrogen of 84 mg/dL, leukocytes at 6.1 × 10^3^/mm^3^, hemoglobin 11 g/dL, platelets 214 ×10^3^/mm^3^, serum albumin 3.9 g/dL, and normal serum electrolytes. Urinalysis showed dipstick proteinuria 1+ and no erythrocytes. The 24-h urine collection had a urine protein-to-creatinine ratio of 109 mg/g.

The immunologic panel was not relevant, as ANA (1:40), anti-dsDNA, anti-PR3, and anti-MPO all tested negative. Serum complement levels were within the normal range (C3: 101 mg/dL, C4: 25 mg/dL). Viral testing was negative for hepatitis B, hepatitis C, and HIV. Cryoglobulins were undetectable. The CT scan showed no tumor lesions, lymph node enlargements, or lytic lesions.

A kidney biopsy was performed to determine the cause of rapidly progressive glomerulonephritis. Under light microscopy, a significant interstitial mononuclear infiltration was observed (Fig. 1).

**Fig. 1.**
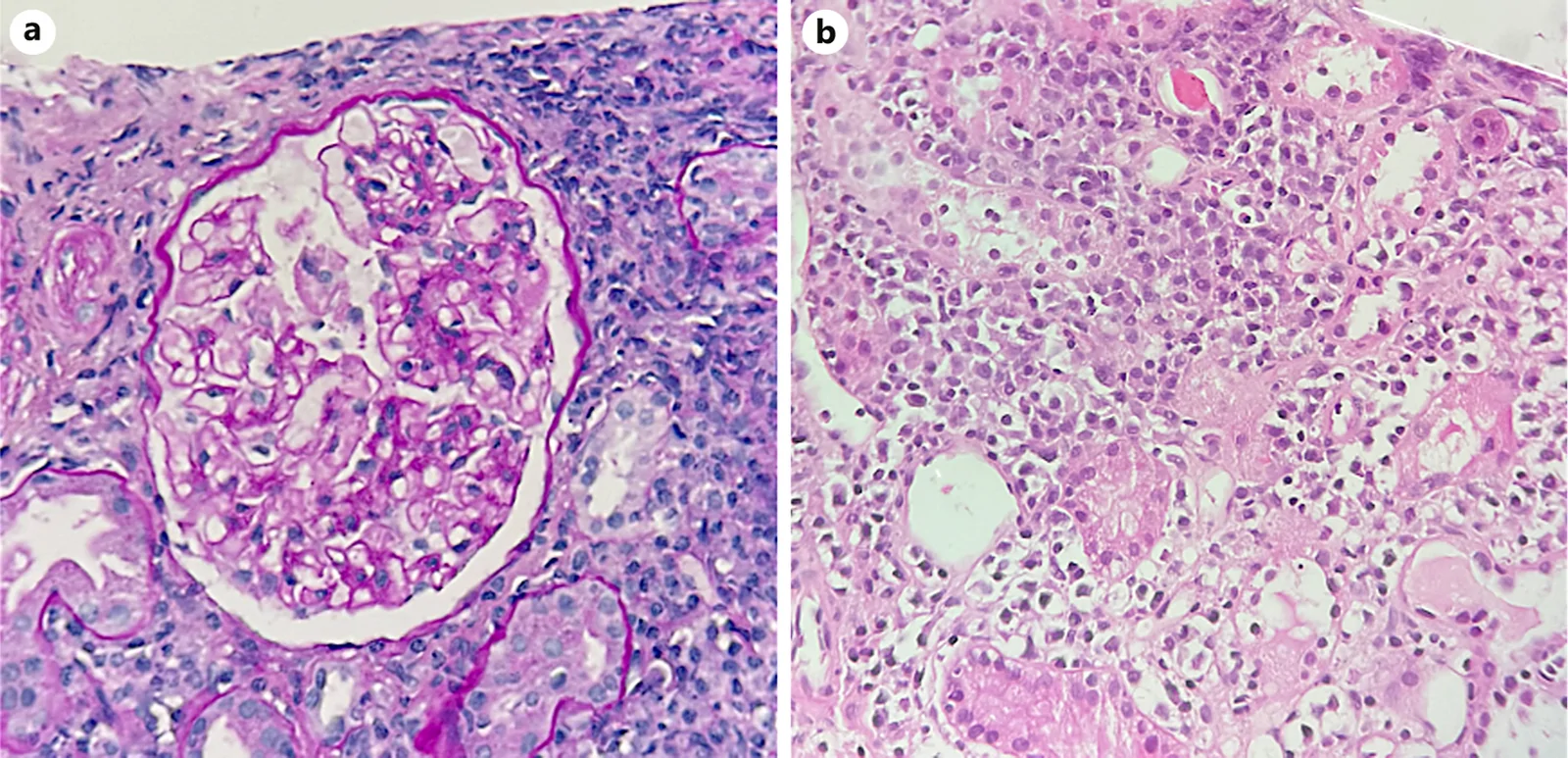
**a** PAS stain showing no relevant macroscopic alterations in the glomerulus. **b** Hematoxylin and eosin stain showing chronic tubulointerstitial inflammatory infiltrate with a predominance of plasma cells.

An immunohistochemistry (IHC) technique revealed a CD138+ interstitial cellular infiltrate, consistent with plasma cells. Additionally, the results showed stains positive for kappa, negative for lambda, IgG, IgG4 subclass, and IgM, while IgA was positive, as identified by immunofluorescence (IF) and IHC (Fig. 2).

**Fig. 2.**
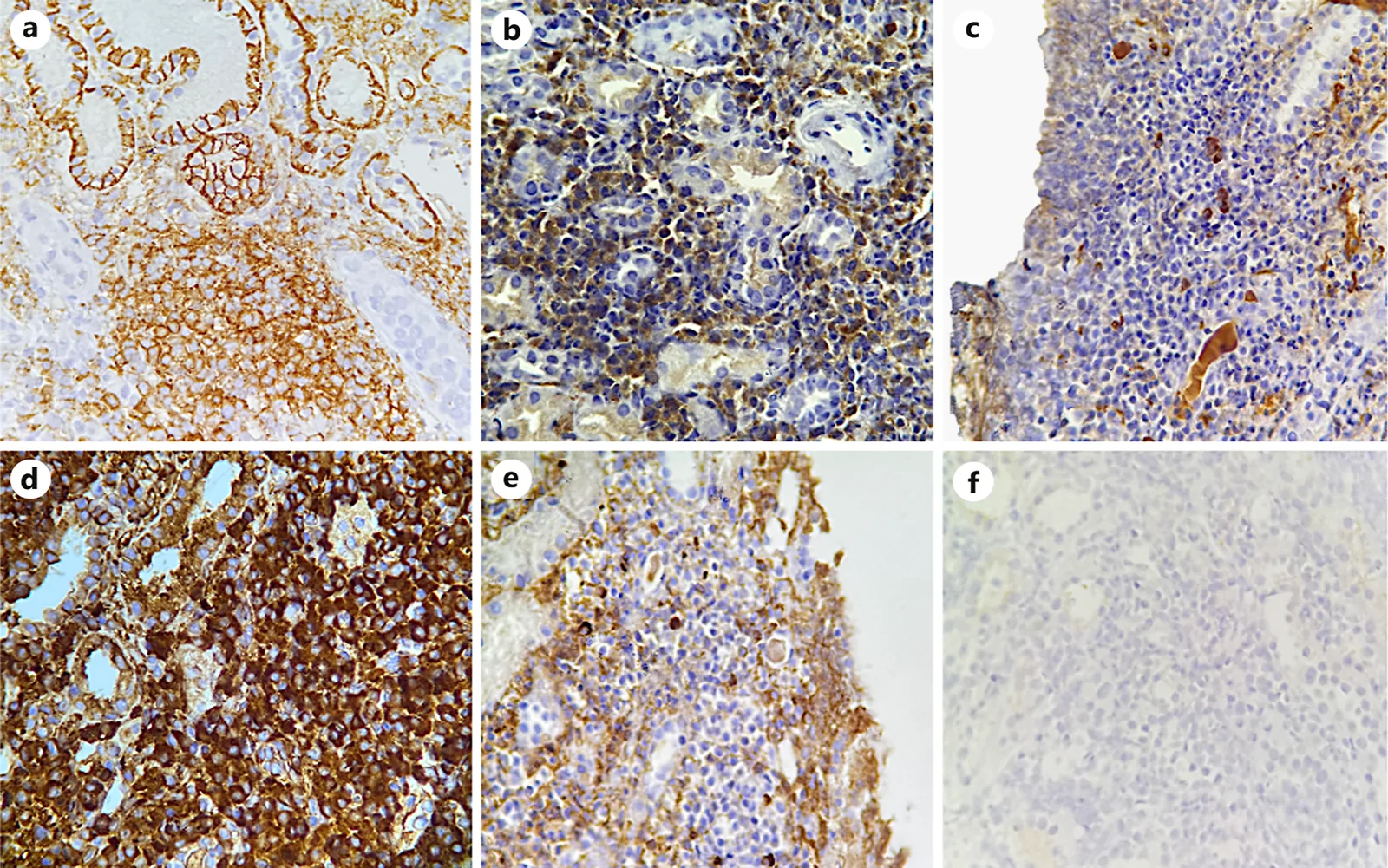
Kidney Immunohistochemical staining (×400) for CD138 (**a**), kappa (**b**), lambda (**c**), IgA (**d**), IgG (**e**), and IgM (**f**).

Based on these findings, we examined plasma cell infiltration in the bone marrow, which showed approximately 50% cellularity, with 3 cell lines showing adequate maturation. The myeloid/erythroid ratio was 3:1, and a few megakaryocytes were observed without morphological changes. CD138-positive cells made up about 3% of the total. Three sequential bone marrow biopsies were performed, all of which revealed no significant plasma cell infiltration. Serum protein electrophoresis and immunofixation revealed a monoclonal spike in IgA and kappa light chain, along with high serum IgA levels (2,283 mg/dL) and an upper-normal limit IgG level (1,556 mg/dL). The serum FLC assay indicated a kappa concentration of 899 mg/dL and a lambda concentration of 217 mg/dL, with a FLC ratio of 4.14. Because there was no plasma cell infiltration in the bone marrow, the hematology team ruled out multiple myeloma. Instead, a diagnosis of monoclonal interstitial nephritis of IgA-kappa plasma cells was established.

In collaboration with the hematology department, it was decided to start chemotherapy (bortezomib + dexamethasone + lenalidomide). At 6 months, the sCr level improved to 1.6 mg/dL, with no proteinuria, urine protein-to-creatinine ratio (<200 mg/g), but high serum IgA (1,920 mg/dL), and no signs of leg edema. Unfortunately, because of the patient’s advanced age and signs of clinical improvement, the patient and their family chose not to proceed with a follow-up renal biopsy. After a 1-year follow-up, eight doses of chemotherapy were given, with stable serum creatinine (sCr) levels (1.7 mg/dL), normal serum IgA (300 mg/dL), and no evidence of progression to multiple myeloma (Fig. 3).

**Fig. 3.**
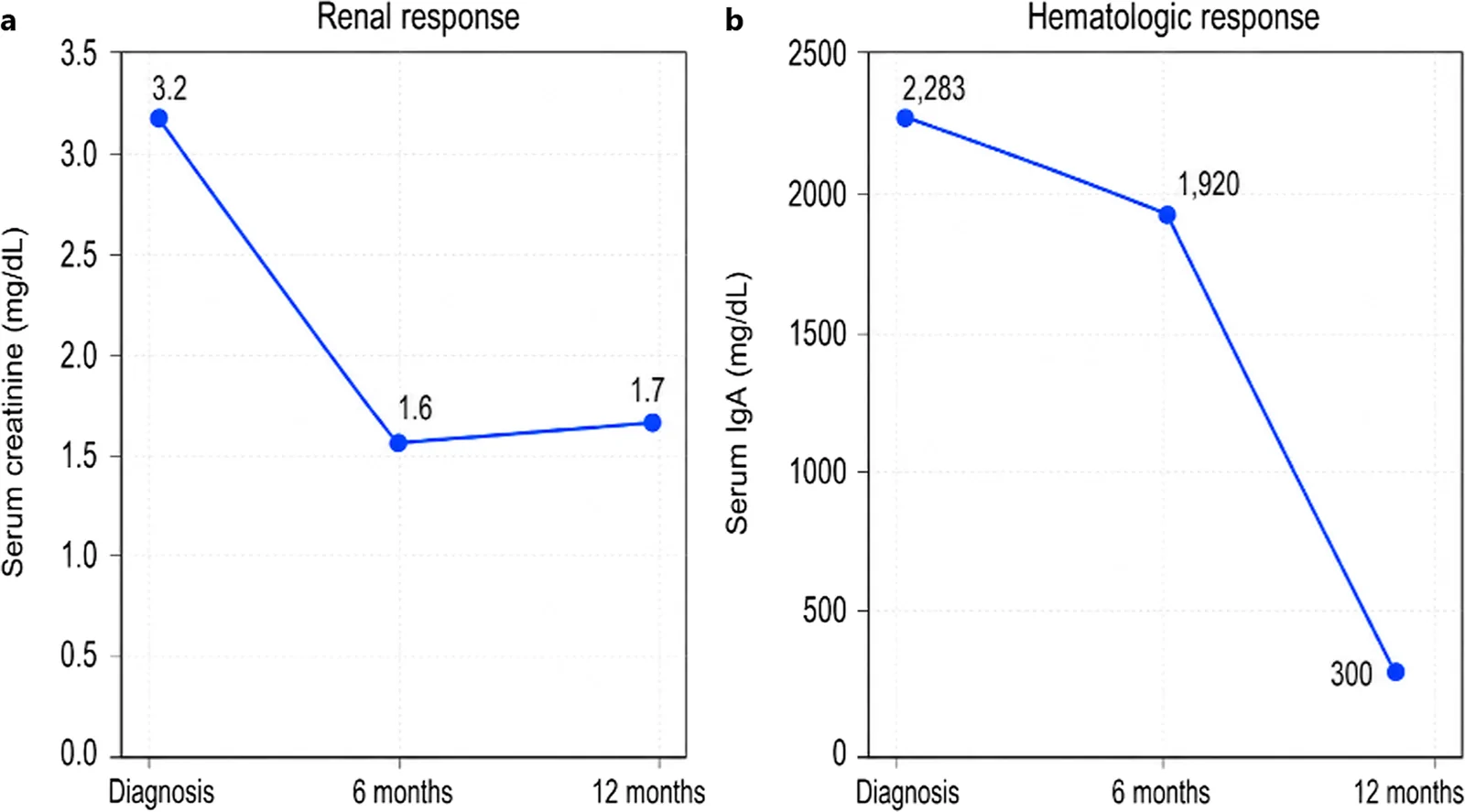
Longitudinal changes in serum creatinine (**a**) and IgA levels (**b**) following plasma cell-directed therapy.

## Discussion

Sjögren syndrome is a chronic autoimmune disease characterized by the gradual infiltration of lymphocytes into the glands, especially the lacrimal and salivary glands [3]. Immune dysregulation in Sjögren syndrome is characterized by an increase in circulating and glandular CD27+ memory B lymphocytes, as demonstrated by the potential effectiveness of long-term memory B-cell depletion strategies in this disease [4].

Long-lived B lymphocytes promote the growth of tertiary lymphoid tissue, enabling affinity maturation, somatic hypermutation, and class switching of immunoglobulins in B lymphocytes [5]. Therefore, increasing the production of chemokines, inflammatory cytokines, and growth factors, facilitated by interleukins such as IL-27, leads to fibrosis, tumor growth, and necrosis [6]. However, the reason for producing an isolated light chain isotype without a detectable pathological clone remains unknown.

Circulating monoclonal immunoglobulins are found in about 20% of patients with Sjögren syndrome, with monoclonal IgM being the most common type [7]. However, as noted above, all reported cases to date have been associated with a monoclonal IgA spike and a clear dominance of kappa light chains, and all have presented with tubulointerstitial nephritis.

The IKMG classification defines three categories based on the composition of deposits and inclusions, as well as findings observed through electron microscopy [8]: nephropathy with organized deposits; nephropathy with unorganized deposits; and nephropathy without monoclonal immunoglobulin deposits. In Sjögren syndrome, tubulointerstitial nephritis is the most frequent renal lesion and may manifest as distal renal tubular acidosis, proximal tubular dysfunction, hypokalemia, nephrogenic diabetes insipidus, or progressive loss of kidney function. In contrast, monoclonal gammopathies may cause tubular injury through proximal tubulopathy with or without crystalline inclusions, Fanconi syndrome, cast nephropathy, and monoclonal immunoglobulin deposition along tubular basement membranes. These lesions are usually related to the physicochemical properties and tubular handling of monoclonal light chains. Our case differs from these established entities because the predominant abnormality was a dense interstitial infiltrate of CD138-positive, IgA-kappa-restricted plasma cells rather than intracellular crystalline deposits, casts, or linear tubular basement membrane deposition. Glomerular lesions are less frequent and usually associated with immune complex deposition, cryoglobulinemia, or lymphoproliferative disorders.

The standard diagnostic approach to renal biopsy – including light microscopy, frozen tissue IF for immunoglobulins and complement, and electron microscopy – is generally sufficient in most cases; however, studies have highlighted the importance of additional techniques such as IgG subclass staining, paraffin-block IF, and IHC in specific situations [9]. IHC and IF have complementary roles in the evaluation of plasma cell-rich tubulointerstitial lesions. IHC allows identification of the infiltrating cell population, particularly through CD138 staining, and may demonstrate intracellular heavy- and light-chain expression in plasma cells. IF is useful for assessing immunoglobulin and light-chain distribution within compartments and for identifying or excluding extracellular monoclonal deposits. Nevertheless, these findings should be interpreted together with serum immunofixation, free light-chain analysis, and bone marrow evaluation.

According to the MGRS classification, except for interstitial amyloidosis and IgM monoclonal glomerulopathy, all histological lesions involve the glomerular and tubular compartments, with no mention of interstitial lesions [1]. Interestingly, evidence shows monoclonal interstitial renal deposits associated with Sjögren syndrome, specifically IgA-kappa plasma cell infiltration.

The first report was described by Kobayashi et al. in Japan in 2006, involving a 49-year-old patient with type 1 renal tubular acidosis and kidney tissue showing abundant interstitial deposits of IgA-kappa lymphocytes [10]. Subsequently, Saglam et al. reported two cases presenting with acute kidney injury, with one case showing interstitial monoclonal IgA-kappa deposits and the other IgA-lambda; both were treated with chemotherapy, leading to improved renal function [11]. Saeki et al. described a 74-year-old woman with progressive renal dysfunction and mild proteinuria, characterized by an FLC ratio of 3.2 and significant interstitial infiltration by CD138+ IgA-kappa plasma cells, who was treated with prednisolone, resulting in substantial improvement in renal function [12]. Lastly, Sonomura et al. reported a 69-year-old man who underwent a kidney biopsy due to acute renal injury, without proteinuria, with an elevated FLC ratio and interstitial deposits of IgA-kappa mononuclear cells; treatment with prednisolone led to partial improvement in kidney function [13].

The predominance of IgA in the reported Sjögren-associated cases is noteworthy. Sjögren syndrome is characterized by sustained B-cell activation, tertiary lymphoid structure formation, somatic hypermutation, and immunoglobulin class switching. Chronic mucosal and systemic immune stimulation could theoretically favor expansion or tissue recruitment of an IgA-committed plasma-cell population. Alternatively, the renal microenvironment may provide chemokines or survival signals that preferentially support these cells [5, 6]. However, this hypothesis remains speculative.

None of the cases reported in the literature to date have progressed to multiple myeloma. Similarly, all patients received immunosuppressive treatment with glucocorticoids, and two-thirds of the patients received plasma cell-directed chemotherapy (Table 1).

**Table 1.** Summary of reported cases of tubulointerstitial nephritis with monoclonal deposits

Author (ref.)	Gender (age)	TI-LC deposition	FLCR	sCr at diagnosis (mg/dL)	Proteinuria at diagnosis (g/day)	sCr after treatment (mg/dL)	Treatment
Present case	Female (83)	IgA	4.1	3.2	UPCR: 109 mg/g	1.7	Bortezomib + lenalidomide + dexamethasone
		Kappa					
Sonomura 2023 [13]	Male (69)	IgA	8.9	2.3	0.10	1.5	Prednisolone
		Kappa					
Naka 2023* [14]	Male (66)	IgA	0.01	2.4	UPCR: 910 mg/g	1.5	Bortezomib + lenalidomide + dexamethasone
		Lambda					
Saeki 2019 [12]	Female (74)	IgA	3.2	1.2	0.20	0.9	Prednisolone
		Kappa					
Saglam 2017 [11]	Female (68)	IgA	0.6	2.2	0.41	1.8	Bortezomib + dexamethasone and Melphalan + prednisolone
		Lambda					
Saglam 2017 [11]	Female (69)	IgA	21.9	2.3	0.17	1.6	Bortezomib + dexamethasone
		Kappa					
Attias 2016* [15]	Male (52)	IgG	7.0	2.4	2.3	2.0	Bortezomib + cyclophosphamide + dexamethasone
		Kappa					

The patients in the studies by Naka et al. [14] and Attias et al. [15] were not related to Sjögren syndrome.

TI-LC, tubulointerstitium light chain deposition; FLCR, free light chain ratio; sCr, serum creatinine; UPCR, urine protein to creatinine ratio.

Tubulointerstitial nephritis, with or without accompanying changes in tubular function such as renal tubular acidosis or Fanconi syndrome, is frequently seen in patients with Sjögren syndrome. Although the current case showed an extensive mononuclear interstitial infiltrate with acute tubular injury, the injury was directly linked to plasma cell infiltration, as demonstrated by the improvement following plasma cell-targeted chemotherapy.

The choice of plasma cell-directed therapy rather than anti-CD20 treatment was based on the phenotype of the pathogenic cell population. The kidney infiltrate was composed predominantly of CD138-positive plasma cells with IgA-kappa restriction, while clinical imaging, bone marrow evaluation, and hematological assessment did not reveal a CD20-positive lymphoid neoplasm.

Another important differential diagnosis to consider is IgG4-related kidney disease (IgG4-KRD), which is characterized by infiltration of IgG4+ plasma cells. It presents with tubulointerstitial nephritis and progressive loss of kidney function, along with mild proteinuria [16]. However, the diagnosis is ruled out due to the absence of deposits positive for the IgG4 subclass and because deposits are usually polytypic in IgG4-KRD.

Another differential diagnosis is the recently described disease, characterized by Takahashi et al. as tubulointerstitial nephritis with IgM plasma cell deposits (IgM PC-TIN), which occurs in patients with Sjögren syndrome and has a high rate of antimitochondrial antibodies (82%); however, in IgM PC-TIN, no monotypic deposits of light chains are detected [17]. Only two cases unrelated to Sjögren syndrome have been reported so far, involving tubulointerstitial nephritis with infiltration of CD138+ plasma cells and monotypic deposits, both of which were associated with acute kidney injury and monoclonal spikes (IgG-kappa and IgA-lambda, respectively). Both patients were treated with plasma cell-directed chemotherapy, showing partial improvement in kidney function [15, 14].

Due to limited reports, this type of tubulointerstitial injury has not been included in the IKMG consensus; however, we believe that this condition qualifies for classification. The damage appears to be directly related to monoclonal immunoglobulin, with histological evidence of plasma cell infiltration showing kappa light chain restriction (or rarely lambda), an abnormal FLC ratio, and signs of improvement following plasma cell-directed chemotherapy.

There is a growing trend to initiate chemotherapy management as early as possible in patients with MGRS. In fact, it is known that the survival prognosis of patients with MGRS resembles that of patients with hematologic malignant lesions [18]. The loss of glomerular filtration rate, FLC ratio, proteinuria, and hematuria are independent factors associated with the presence of MGRS [2, 18]; therefore, they should be treated to prevent adverse outcomes.

The clinical course of the previously reported cases suggests that both immunosuppressive therapy and plasma cell-directed chemotherapy may improve kidney outcomes. In patients treated with glucocorticoids alone, serum creatinine generally decreased, indicating that suppression of the inflammatory tubulointerstitial component may be beneficial (Table 1). Conversely, bortezomib-based regimens were associated with partial renal recovery or stabilization and may additionally reduce the pathogenic monoclonal immunoglobulin-producing clone. Even in patients where the pathological clone cannot be detected, empirical management with lymphocytic-clone or plasma cell-directed therapy has been considered, as in PGNMID, with some evidence supporting this strategy [19].

In conclusion, monoclonal interstitial nephritis with IgA-kappa plasma cells is a relatively new condition in patients with Sjögren syndrome, with few cases reported so far. It may represent a novel MGRS-associated lesion, especially when there is convincing clinicopathological concordance. In the present case, the interstitial infiltrate showed IgA-kappa restriction; the circulating monoclonal protein had the same isotype; the serum free light-chain ratio was abnormal; and repeated bone marrow examinations did not demonstrate myeloma-level plasma-cell infiltration. Furthermore, kidney function improved with plasma cell-directed therapy. These findings support a causal relationship between the small plasma-cell clone and kidney injury. Therefore, treatment with glucocorticoids and/or plasma cell-targeted chemotherapy could improve outcomes in this group of patients.

## Statement of Ethics

Ethics approval for this human study was waived by Comité de Ética e Investigación del Centro Médico Nacional “20 de Noviembre.” Written informed consent was obtained from the patient to publish the medical case details and accompanying images. The authors have completed the CARE Checklist for this case report, attached as online supplementary material (for all online suppl. material, see https://doi.org/10.1159/cnd/abxag004).

## Conflict of Interest Statement

The authors declare that they have no conflicts of interest.

## Funding Sources

This study was not supported by any sponsor or funder.

## Author Contributions

A.S.M.: supervision, conceptualization, and writing – original draft. R.G.N.A., C.H.J.S., C.L.M., and T.G.C.: writing – original draft. H.H.C.R. and M.M.C.: documenting research data.

## Data Availability

All data generated or analyzed during this study are included in this article. Further inquiries can be directed to the corresponding authors.
